# A Rapidly Progressing Carotid Body Tumor: A Case Report

**DOI:** 10.7759/cureus.43654

**Published:** 2023-08-17

**Authors:** Colby Kihara, Siddharth Patel, Roger Moss

**Affiliations:** 1 Research, Alabama College of Osteopathic Medicine, Dothan, USA; 2 Internal Medicine, Decatur Morgan Hospital, Decatur, USA; 3 Family Medicine, Decatur Morgan Hospital, Decatur, USA

**Keywords:** general and vascular surgery, coronary computed tomography angiogram (cta), pulsatile neck mass, head and neck paraganglioma, carotid body tumor

## Abstract

Carotid body tumors (CBTs) are a rare form of paragangliomas that often arise near the carotid bifurcation. They are typically slow growing and often asymptomatic. We report a case of a CBT in a 70-year-old man, who presented with a firm, painless, pulsatile neck mass that rapidly increased in size over the course of two months. The diagnosis was suspected based on the patient’s clinical history and physical examination. The diagnosis was confirmed with CT angiography (CTA). The tumor was nonfunctioning based on normal urinary-free catecholamines, vanillylmandelic acid (VMA), and metanephrines levels. The patient then underwent a CT scan of his thorax, abdomen, and pelvis which did not detect any metastatic spread. The patient was referred to a tertiary vascular surgery center for definitive treatment. Our aim in presenting this case is to increase awareness of this rare type of paraganglioma with the hope of increasing early intervention and improving outcomes.

## Introduction

The carotid body is a sensory organ typically 2 to 6 mm in diameter located near the bifurcation of the common carotid artery bilaterally [1]. The carotid body is the largest collection of paraganglia in the head and neck and can be the site for carotid body tumors (CBTs). CBTs, also known as chemodectomas, are typically slow-growing, extra-adrenal tumors of neuroendocrine origin which arise from either sympathetic or parasympathetic paraganglia throughout the body [2,3]. CBTs are rare, highly vascular tumors that comprise only 0.3% of all paragangliomas but 60% of head and neck paragangliomas. The majority of CBTs are benign and nonfunctioning. However, functioning CBTs may produce symptoms of headache, excessive sweating, and palpitations which should be evaluated with 24-hour urine collection for catecholamines, metanephrines, and vanillylmandelic acid (VMA). Furthermore, malignant CBTs can invade local cranial nerves such as the hypoglossal nerve, glossopharyngeal nerve, vagus nerve, or sympathetic chain and cause Horner syndrome [4]. The reported incidence of CBTs is roughly 1-2 per 100,000 people [5].

The diagnosis of CBTs is based on clinical history, physical examination, and radiological imaging such as ultrasound, CT scan, and MRI [6]. Early surgical excision is considered the treatment of choice and is often preceded by preoperative embolization of feeding arteries. However, in cases where the surgical risk is high or surgery is not an option, radiotherapy may also be explored as a means of treatment [7]. We present a case of a CBT which presented as an asymptomatic neck mass.

## Case presentation

A 70-year-old man with a 10-year history of asthma, chronic headaches, hypertension, hemorrhoids, and hyperlipidemia presented to the clinic with a newly discovered mass on the left side of his neck. The patient first noticed the mass a few months prior and noted that it has been progressively increasing in size since he first noticed it. The patient is a former smoker with a 30-pack-year history. The patient denied any recent illness, pain, discomfort, neck stiffness, difficulty hearing or swallowing, tinnitus, fever, night sweats, weight changes, shaking chills, dizziness, lightheadedness, weakness, fatigue, hoarseness, blurry vision, flushing, diaphoresis, palpitations, nausea, vomiting, diarrhea, or constipation.

**Figure 1:**
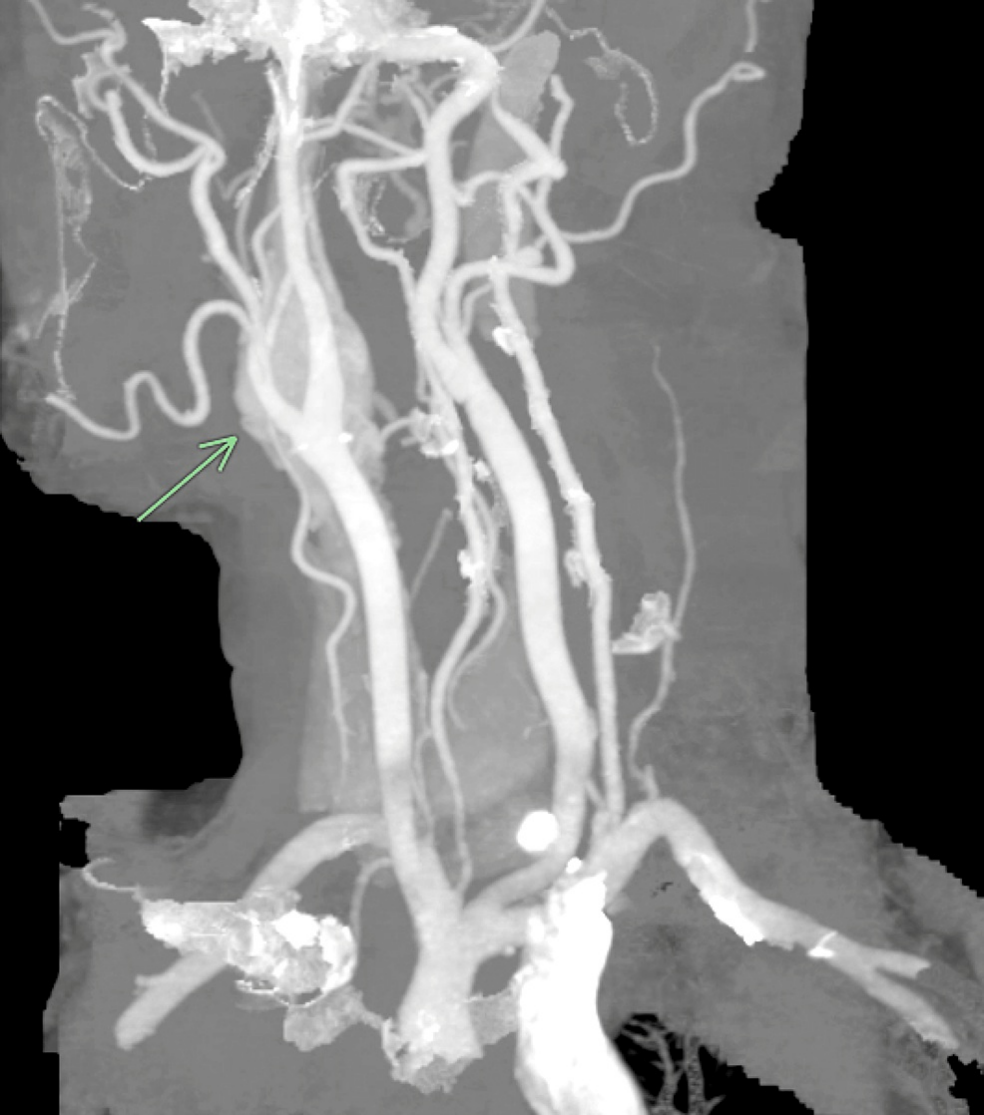
CTA of the neck demonstrating a left neck mass splaying the internal and external carotid arteries known as a Lyre sign

**Figure 2:**
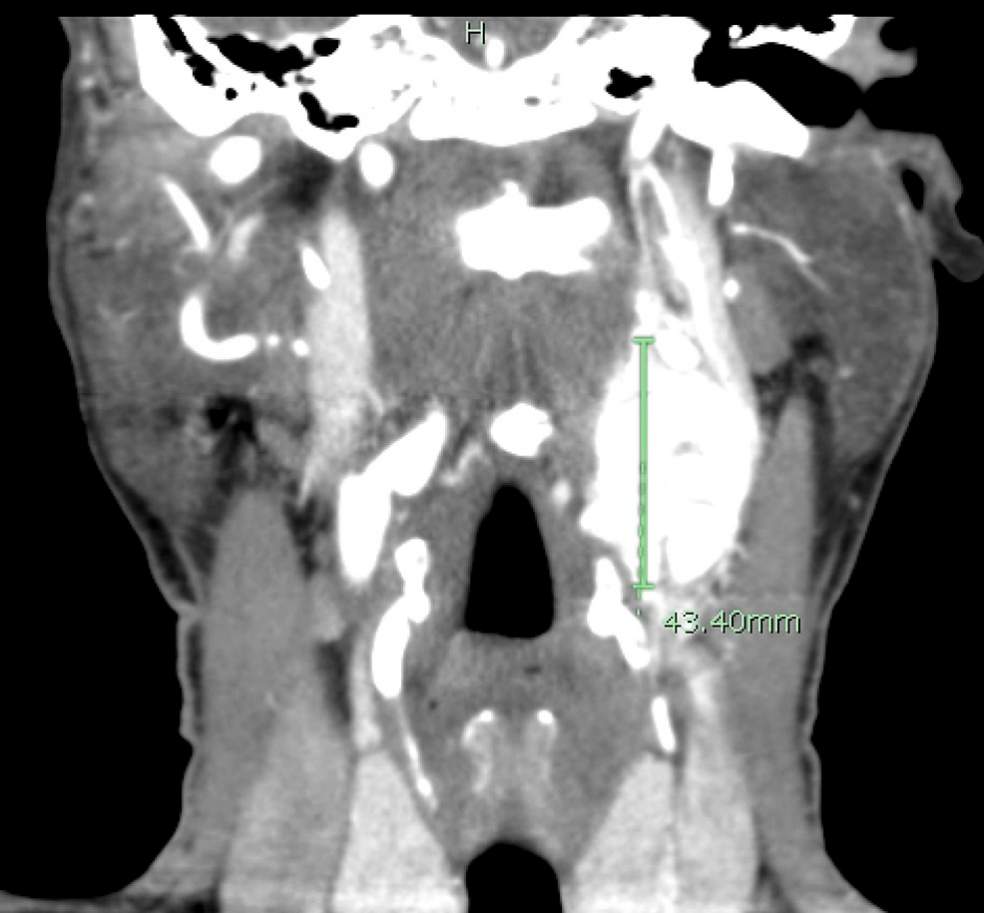
Coronal section of a neck CTA showing a left neck mass measuring 4.3 cm craniocaudal measurement

**Figure 3:**
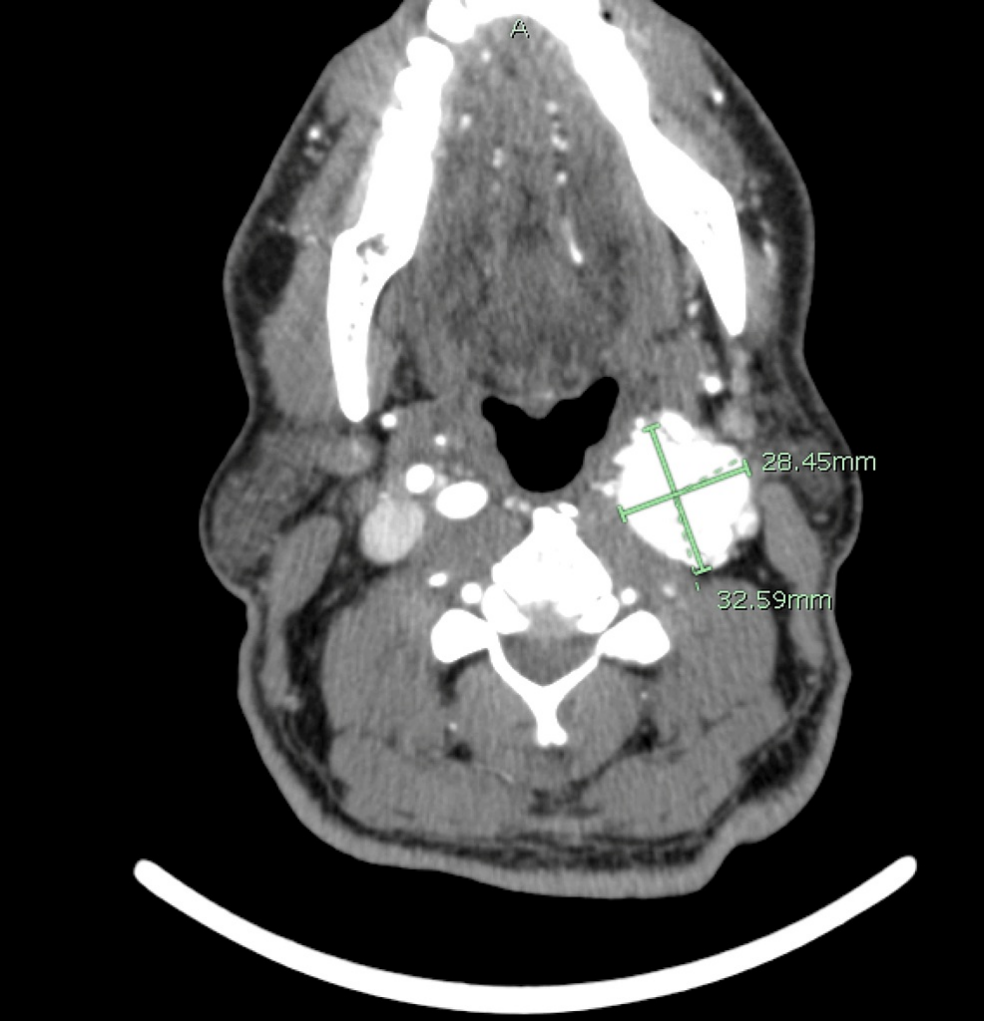
A transverse head and neck CTA showing a left neck mass measuring 3.3 x 2.8 cm in anterior-posterior and lateral measurements

On clinical exam, a roughly 4 x 2 cm mass was palpated on the left side of the patient’s neck near the angle of the mandible. The mass was firm, slightly mobile side-to-side, and pulsatile. The remainder of the exam was unremarkable. CT angiogram of the head and neck found a large, hypervascular, hyperenhancing mass at the left carotid bifurcation which caused splaying of the internal and external carotid arteries. This mass measured 3.3 x 2.8 x 4.3 cm in anterior-posterior, lateral, and craniocaudal measurements and was diagnosed to be a paraganglioma (Figures 1-3). No adenopathy was noted and the thyroid gland appeared normal. The patient then had his urine collected to assess for free catecholamines, VMA, and metanephrines all of which were found to be within normal limits. The patient then underwent a CT scan with oral and IV contrast of his thorax, abdomen, and pelvis to assess for metastatic spread. The CT scan found no discrete metastatic disease.

## Discussion

The carotid body was first described by Von Haller in 1743 and can serve as the growth site for CBTs. CBTs are rare, slow-growing, neuroendocrine tumors that can undergo malignant transformation [6]. While the exact pathogenesis remains unknown, it is thought that genetic factors and hypoxia play a role in the formation of CBTs. The most common mutation associated with the formation of CBTs is a succinate dehydrogenase mutation. Hypoxia appears to enhance the succinate dehydrogenase mutation [8]. CBTs are a type of paraganglioma derived from embryonic neural crest cells. Paragangliomas are rare tumors with an incidence of roughly 1-2 per 100,000. Of these, roughly 3% are located within the head and neck with the majority being CBTs, vagal paragangliomas, or glomus jugulare tumors [9]. While CBTs appear in a wide variety of ages, most CBTs occur in patients between 35.9 and 71.1 years old with the majority (60.4%) being female [10].

CBTs typically present as painless, asymptomatic, slowly enlarging lateral neck masses. On physical exam, they tend to be found near the border of the sternocleidomastoid muscle and move more freely horizontally than vertically, known as a Fontaine’s sign. This is due to the adherence of the tumor to the carotid artery. As such, these tumors often have a pulsatile character and can present with a carotid bruit. Furthermore, if the lesion is large enough and/or malignant, it can, on rare occasions, produce neurological deficits as it encroaches or invades surrounding cranial nerves or blood vessels [11].

CBTs are often discovered on physical exams or incidentally in imaging studies. Digital subtraction angiography is the gold standard for diagnosing CBTs which will show a splaying of the internal and external carotid arteries known as a Lyre sign. However, while digital subtraction angiography is the current diagnostic imaging of choice, it should be noted that color Doppler ultrasound is a simple, non-invasive means of imaging that has a relatively high sensitivity and specificity for CBTs [6].

In 1971, Shamblin et al. introduced a way to classify CBTs as an aid to help predict the prognosis and difficulty of surgical resection of CBTs. The research group classified the tumors in relation to the carotid vessels. Group 1 tumors are small and have a minimal attachment to the carotid vessels; therefore, surgical excision should be carried out without difficulty. Group 2 tumors are larger and show moderate attachment to the carotid arteries but do not encase the vessels. As such, these tumors may be more difficult to excise. Group 3 tumors are usually large and fully encase the carotid arteries. Excision of this group is associated with the most complications, and vessel replacement should be considered [12]. In 2005, Luna-Ortiz et al. argued that the Shamblin criteria must be modified to better predict surgical difficulties and complications. As such, the group of researchers proposed adding a fourth group splitting the original Group 3 into groups 3A and 3B where 3A would represent the original Group 3 and 3B would now encompass those tumors that both circumferentially encased the vessel as well as infiltrated it [13].

While most CBTs tend to be slow growing with a median growth rate of 1.0 mm per year [14], here we present a case where the tumor went from being barely palpable to over 4 cm in the span of a few months. While most CBTs are benign, it is estimated that 3-10% have malignant potential making early diagnoses and surgical treatment imperative [15]. Furthermore, if the CBT grows to 5 cm or larger, the mortality after surgical intervention can be as high as 3% [16]. Surgical excision of CBTs began in 1880 and was associated with high rates of complications such as nerve damage, stroke, hemorrhage, and death [10]. Today, however, with enhancements in imaging and selective embolization techniques, cranial nerve injury, recurrence, morbidity, and mortality are very low, making surgical excision a very safe and effective modality in the treatment of CBTs [7].

## Conclusions

While the differential diagnosis for a neck mass can be broad and include pathologies such as hematomas, schwannomas, carotid artery aneurysms and pseudoaneurysms, head and neck carcinoma, cyst, and many more, we hope our case report serves to highlight the importance of having CBTs on that differential list. Due to early diagnostic imaging, our patient was able to have his CBT detected before it reached 5 cm, which is the size associated with an increased risk of surgical complications and death. Additionally, due to a CBT’s potential for malignancy, this case report serves to highlight the need for early diagnostic imaging in patients with a lateral, asymptomatic neck mass.
